# *Chromobacterium violaceum* infection in chronic granulomatous disease: a case report and review of the literature

**DOI:** 10.1099/jmmcr.0.005084

**Published:** 2017-01-31

**Authors:** Zaal Meher-Homji, Rekha Pai Mangalore, Paul D. R. Johnson, Kyra Y. L. Chua

**Affiliations:** ^1^​ Department of Infectious Diseases, Austin Health, Heidelberg, VIC 3084, Australia; ^2^​ Department of Microbiology, Austin Health, Heidelberg, VIC 3084, Australia

**Keywords:** *Chromobacterium violaceum*, Lemierre's syndrome, meropenem, trimethoprim/sulfamethoxazole

## Abstract

**Introduction.** *Chromobacterium violaceum* is an opportunistic human pathogen, associated with significant mortality, and has been reported in patients with chronic granulomatous disease (CGD), a genetic condition causing impaired phagocytosis.

**Case presentation.** A 28-year-old man with a history of CGD presented with fever, pharyngitis, cervical lymphadenopathy and internal jugular vein thrombosis, following travel to the rural Solomon Islands. *C. violaceum* was recovered from his blood. The patient recovered after treatment with meropenem and trimethoprim/sulfamethoxazole.

**Conclusion.** To the best of our knowledge, this is the first case report of internal jugular vein thrombophlebitis (Lemierre’s syndrome) caused by *C. violaceum* in a patient with CGD. A review of the literature demonstrated that the diagnosis of *C. violaceum* preceded the diagnosis of CGD in the majority of cases. This case emphasizes the importance of this organism in patients with CGD who live in or visit tropical areas.

## Abbreviations

CGD, chronic granulomatous disease; CT, computed tomography; TMP/SMX, trimethoprim/sulfamethoxazole.

## Introduction

*Chromobacterium violaceum* is a motile facultatively anaerobic Gram-negative bacillus, found in the water and soil of tropical areas [1]. It is catalase and oxidase positive, often producing β-haemolytic colonies and a characteristic violet pigment, violacein. It is an uncommon human pathogen, usually acquired from a break in the skin with subsequent exposure to soil or water. The resulting infection is usually severe, with a wide spectrum of infections described including bacteraemia, and skin and visceral abscesses. Historical cases report high mortality rates of 53 % [2]; however, in a more recent case series of 28 patients from the Northern Territory of Australia the mortality rate (7.1 %) was far lower than previously described [3].

Chronic granulomatous disease (CGD) is a heterogeneous genetic disease of phagocyte NADPH oxidase, which results in impaired respiratory burst and production of hydrogen peroxide and other oxygen products, leading to a predisposition to infection with catalase-positive organisms [4]. Patients typically present with recurrent bacterial and fungal infections, failure to thrive or poor wound healing. The diagnosis is made by neutrophil function testing and genetic testing. Commonly reported infections include *Aspergillus* species, *Staphylococcus aureus*, *Burkholderia cepacia*, *Serratia marcescens* and *Nocardia* species [4].

Previous case series have demonstrated *C. violaceum* bacteraemia in patients with CGD [5, 6]. However, to the best of our knowledge, this is the first case report of *C. violaceum* causing internal jugular vein thrombophlebitis or Lemierre’s syndrome in a patient with CGD. We summarize the reported cases of *C. violaceum* in CGD patients, including this current case.

## Case report

A 28-year-old man presented with fever, pharyngitis and cervical lymphadenopathy after returning from the Solomon Islands 2 days prior to his presentation. His past medical history was notable for CGD, which was diagnosed as a child following presentations with recurrent pneumonia and sinusitis. He was treated for pulmonary tuberculosis at age 4 years, but subsequently presented 2 years prior to his current admission with left upper lobe cavitary lung lesions and was retreated for culture-negative tuberculosis with rifampicin, isoniazid, pyrazinamide and ethambutol.

One month prior to his current admission, the patient travelled to rural areas of the Solomon Islands. The patient was not taking his antibiotic prophylaxis at the time. He described both fresh and salt-water contact with a number of lacerations on his feet, although none of these became inflamed or infected. One week prior to return to Australia, he became unwell with a prominent sore throat, fever and subsequent neck pain. After arrival in Australia, he presented to our institution.

On examination the patient was febrile (39 °C), had significant hypotension [systolic blood pressure 70 mmHg (9.3 kPa)], a pulse rate of 160 beats min^−1^, a respiratory rate of 24 breaths min^−1^ and an oxygen saturation of 93 % on room air. His neck was asymmetrically swollen on the left, without any fluctuance. The oropharynx was erythematous and oedematous without a visible collection or any evidence of airway compromise. The lacerations on his feet were all well healed, with no evidence of inflammation.

## Investigations

**Fig. 1.**
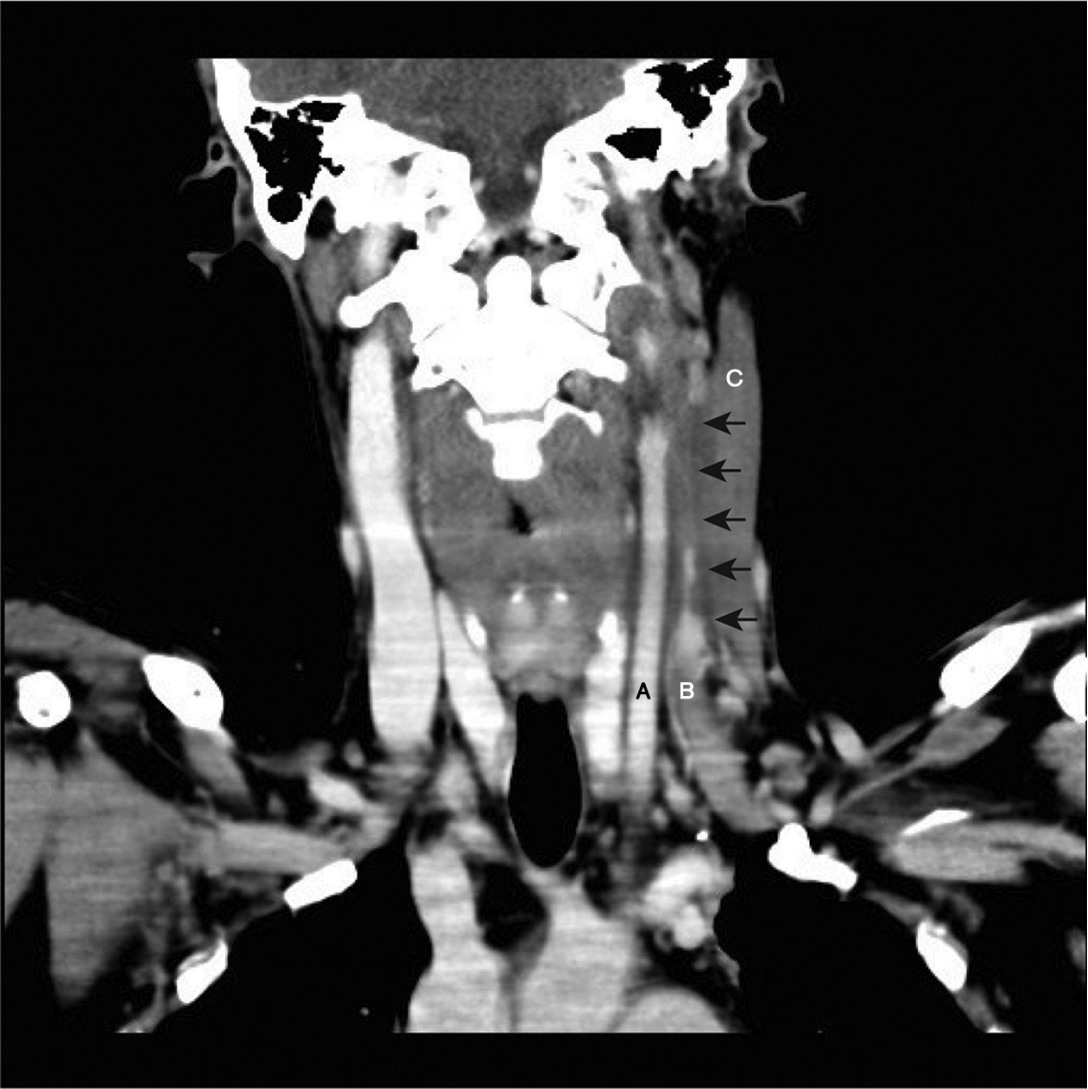
Post-contrast CT scan of the neck showing the left common carotid artery (A) and left internal jugular vein (B). Arrows indicate the extent of the thrombosis and the filling defect of the left internal jugular vein at the level of the thyroid cartilage, extending superiorly to the base of skull. There was also fusiform thickening of the left sternocleidomastoid muscle (C) without a discrete collection.

Initial tests revealed an elevated total white cell count (17×10^9^ cells l^−1^) and neutrophils (10.2×10^9^ cells l^−1^), C-reactive protein of 286 mg l^−1^ and a lactate level of 3.2 mmol l^−1^. A chest X-ray demonstrated a new opacity in his left lower lobe. Arterial blood gases demonstrated pH 7.44, pO_2_65 mmHg (8.7 kPa) and pCO_2_32 mmHg (4.3 kPa). Ultrasonography of his neck revealed an occlusive thrombus of the left mid to upper internal jugular vein. This was subsequently confirmed with computed tomography (CT), which demonstrated thrombosis of the left internal jugular vein at the level of the thyroid cartilage, extending superiorly to the base of skull (Fig. 1). The CT imaging also demonstrated centrally necrotic consolidation in the anterior left lower lobe and multiple ill-defined pulmonary nodules. These clinical and imaging findings were consistent with Lemierre’s syndrome, that is, internal jugular vein thrombophlebitis.

## Diagnosis

Two sets of blood cultures (both aerobic and anaerobic bottles) flagged positive after 1 day of incubation with a motile Gram-negative bacillus. The organism grown was oxidase positive (*N*,*N*,*N′*,*N′*-tetramethyl-*p*-phenylenediamine dihydrochloride; BD BBL) with β-haemolytic colonies that had a distinctive deep purple pigment. It was identified using matrix-associated laser desorption ionization-time of flight MS using the VITEK MS platform (bioMérieux) as *C. violaceum* (identification 99.9 % probability). A VITEK 2 identification by GN card was also in agreement (bionumber 4067000140541210; bioMérieux). MICs by Etest (bioMérieux) on Mueller–Hinton agar (Oxoid) were: ceftazidime, >256 µg ml^−1^; cefepime, 1.5 µg ml^−1^; piperacillin/tazobactam, 16/4 µg ml^−1^; meropenem, 0.38 µg ml^−1^; aztreonam, 6 µg ml^−1^; gentamicin, 1.5 µg ml^−1^; tobramycin, 1 µg ml^−1^; amikacin, 6 µg ml^−1^; trimethoprim/sulfamethoxazole (TMP/SMX), 0.032 µg ml^−1^; chloramphenicol, 2 µg ml^−1^; doxycycline, 0.75 µg ml^−1^; and ciprofloxacin, 0.012 µg ml^−1^.

## Treatment

The initial antibiotics commenced were ceftriaxone (2 g, twelve hourly), metronidazole (500 mg, eight hourly) and flucloxacillin (2 g, six hourly). This was subsequently changed to meropenem (1 g, eight hourly) on day 1, when the Gram-negative bacillus was identified as *C. violaceum*. The patient also received inotropic and respiratory support by mechanical ventilation. He was commenced on heparin anticoagulation treatment (as per the activated partial thromboplastin time), because repeat ultrasonography demonstrated further extension of the internal jugular vein thrombus.

Despite clinical improvement with cessation of inotropic and ventilatory support, the patient had a recrudescence of fever on the seventh day of treatment with meropenem. No other sources of infection were identified, neither was *C. violaceum* re-isolated. On the presumption that meropenem could have caused a drug fever, this agent was replaced with TMP/SMX (160/800 mg, twice daily). The patient gradually defervesced and was subsequently discharged home after 16 days in hospital, on anticoagulation treatment with rivaroxaban (20 mg, once daily).

## Outcome and follow-up

The patient remained well at 8 months post-discharge from hospital. He completed a 12week course of treatment with TMP/SMX (160/800 mg, twice daily). A prolonged course was chosen because of the recurrences described in the literature in patients with CGD [7]. Following this treatment course, the patient was then on antibiotic prophylaxis for his CGD with TMP/SMX (160/800 mg, once daily) and itraconazole (150 mg, once daily). Anticoagulation therapy was discontinued following complete resolution of the internal jugular vein thrombus on imaging.

## Discussion

Fewer than 200 cases of human infection with *C. violaceum* have been described in the literature, with the majority of cases acquired in South-East Asia, the Indian subcontinent, the South-Eastern USA and Northern Australia [2, 3]. The association between CGD and *C. violaceum* has been observed by others [5–17]. Clinical isolates of *C. violaceum* are highly virulent when administered to p47*phox*−/− mice with defective NADPH oxidase compared to controls, with a mean of 13 organisms causing 90 % mortality [18]. *In vitro* studies by the same authors showed *C. violaceum* isolates were sensitive to both hydrogen peroxide and exogenous reactive nitrogen intermediates. Defence against *C. violaceum* is likely to rely on NADPH oxidase function that is lacking in patients with CGD {18}.

Table 1 summarises the 15 cases of *C. violaceum* in patients with a preceding or subsequent diagnosis of CGD. The median age was 13 years (range 1–28 years). A total of 60 % (9/15) cases were acquired and described in the USA, with the majority from either Florida or North Carolina. The majority of patients (10/15, 67 %) were only diagnosed with CGD subsequent to being infected with *C. violaceum*. Interestingly, 43 % (6/14, unknown for 1 case) of these patients did not report a preceding history of multiple bacterial/fungal infections prior to infection with *C. violaceum*. In contrast to a larger case series of non-CGD patients [2], the majority of CGD patients (14/15, 93 %) infected with *C. violaceum* survived. However, this is probably a selection bias, because in most cases, the diagnosis of CGD was made after presentation with *C. violaceum* infection, and the patients who succumbed to fatal *C. violaceum* infection may not have had the opportunity for testing for CGD. In addition, diagnostic testing for CGD, which requires neutrophil function testing and often subsequent genetic testing, may not have been widely available [3]. The true incidence of CGD in patients with *C. violaceum* infection may be higher. As is described in Table 1, most cases of *C. violaceum* and CGD presented with severe disease, rather than being limited to the skin and soft-tissue infections that have been described in immunocompetent patients [3].

A variety of treatment regimens has been utilized (Table 1), similar to that described in non-CGD patients, although in the more recently described cases fluoroquinolones have been frequently employed. An *in vitro* study by Aldridge and colleagues demonstrated that these were the most-active agents against *C. violaceum* [19]. The isolate of *C. violaceum* from our patient had similar susceptibility results to those in previously described reports, with low MICs to fluoroquinolones, TMP/SMX and carbapenems [2]. TMP/SMX was chosen in this instance for convenience, as the same antibiotic is used, following completion of treatment, in lowered doses for longer-term prophylaxis against infection in CGD patients.

**Table 1.** Reported cases of *C. violaceum* in patients with known or subsequently diagnosed CGD F, female; M, male; ARDS, acute respiratory distress syndrome.

**Case no.**	**Reference**	**Age (years)**	**Sex**	**Country/area acquired**	**CGD status at time of *C. violaceum***	**Previous infections**	***C. violaceum* clinical syndrome**	**Treatment**	**Outcome**
1	[6]	28	M	USA (Florida)	Known	*Mycobacterium fortuitum*	Septicaemia, cutaneous pustules, lymphadenitis, spleen/liver and lung abscesses	Gentamicin and chloramphenicol for 8 days	Died
2	[8]	4	M	USA (Florida)	Unknown	Nil	Bacteraemia, osteomyelitis	Gentamicin and TMP/SMX plus drainage	Survived
3	[6]	15	M	USA (Florida)	Known	Fever of unknown origin	Bacteraemia, lung abscesses	Gentamicin and chloramphenicol for 3 weeks	Survived
4	[9]	13	M	Brazil	Unknown	Nil	Multiple skin abscesses, partially treated, recurrent right inguinal abscess 2 years later	Chloramphenicol and TMP/SMX for 21 days	Survived
5	[10]	9	M	USA (Florida)	Known	Nil	Purulent forearm lesion with subsequent periorbital and orbital cellulitis	Carbenicillin and chloramphenicol	Survived
**6**	[11]	1.5	M	USA (Florida)	Unknown	Pneumonia	Pustule with axillary lymphadenitis, hepatosplenomegaly	Chloramphenicol	Survived
**7**	[12]	3	M	Australia (Northern Territory)	Unknown	Recurrent skin sepsis	Septicaemia, chest wall furuncle and lymphadenitis	TMP/SMX for 5 weeks	Survived
8	[13]	13	M	USA (North Carolina)	Unknown	Unknown	Fulminant sepsis with multi-organ failure, ARDS	Unknown	Survived
9	[5]	3	M	Thailand	Unknown	Nil	Lung, liver and splenic abscesses, progressing over 4.5 months	Meropenem, then ciprofloxacin (subsequently lost to follow-up)	Survived
10	[14]	13	M	USA (North Carolina)	Unknown	Nil	Ecthyma gangrenosum, septicaemia	Chloramphenicol, ciprofloxacin and gentamicin for 9 weeks	Survived
11	[7]	20	M	Malaysia	Known	*C. violaceum* infected wounds	Recurrent sepsis due to *C. violaceum*, left forearm pustule with liver and splenic abscesses	Ciprofloxacin and TMP/SMX for 2 weeks	Survived
12	[15]	14	M	USA (North Carolina)	Unknown	Cat-scratch disease	Facial necrotizing fasciitis	Imipenem and gentamicin, with limited nasal and oral resection, for 6 weeks	Survived
13	[16]	11	M	USA (Alabama)	Unknown	*Nocardia* submental abscess	Gluteal abscess, sepsis and ARDS, septic emboli to lungs and liver	Meropenem and ciprofloxacin for 6 months	Survived
14	[17]	11	F	India	Unknown	Nil	Ecthyma gangrenosum, septicaemia, liver and splenic abscesses	Piperacillin/tazobactam, gentamicin then ciprofloxacin for 10 weeks	Survived
15	This study	28	M	Solomon Islands	Known	*Mycobacterium tuberculosis*	Lemierre's syndrome (suppurative jugular thrombophlebitis)	TMP/SMX for 12 weeks	Survived

The current case described herein differed from cases in the existing literature in that there were no skin and soft-tissue infections uncovered. With the history of foot lacerations and water exposure, it is possible that haematogenous seeding of the internal jugular vein occurred without an overt local infection. Alternatively, oropharyngeal spread by breach of the oral mucosa may have been possible, as has been described with other cases of Lemierre’s syndrome caused by different organisms [20]. Although there were pulmonary metastatic septic lesions, no surgical debridement of the neck space, mediastinum or lungs was required. Roberts and colleagues described a case of *C. violaceum* also resulting in deep neck space infection and internal jugular vein thrombosis in an otherwise healthy man [21]. In this instance, the neck space infection was preceded by a lower limb infection following a coral cut in Thailand.

In conclusion, patients with CGD are susceptible to a small number of catalase-positive pathogens, including *C. violaceum*. Given the association, it may be prudent to screen younger patients who present with *C. violaceum* infection with neutrophil function testing, as this may be the ‘sentinel infection’ that prompts the diagnosis of CGD. This case also highlights the importance of pre-travel counselling in patients with CGD and, where possible, compliance with antibiotic prophylaxis should be encouraged. *C. violaceum* has also been reported in immunocompetent hosts, and infection is severe resulting in septicaemia, intensive care unit admissions and high mortality rates. The rate of CGD in patients with *C. violaceum* is likely under-reported due to high mortality and the inability to perform neutrophil function testing. Whilst *C. violaceum* is a rare entity, typical features described in the majority of case reports include skin and/or visceral abscesses. However, clinicians must be mindful that some cases present atypically and prompt microbiological diagnosis is key to commencing appropriate antimicrobials.
